# Testing the effects of sensory stimulation as a collateral-based therapeutic for ischemic stroke in C57BL/6J and CD1 mouse strains

**DOI:** 10.1371/journal.pone.0183909

**Published:** 2017-09-13

**Authors:** Aneeka M. Hancock, Ron D. Frostig

**Affiliations:** 1 Department of Neurobiology and Behavior, University of California Irvine, Irvine, California, United States of America; 2 Center for the Neurobiology of Learning and Memory, University of California Irvine, Irvine, California, United States of America; 3 Department of Biomedical Engineering, University of California Irvine, Irvine, California, United States of America; Fraunhofer Research Institution of Marine Biotechnology, GERMANY

## Abstract

Utilizing a rat model of ischemic stroke, we have previously shown that sensory stimulation can completely protect rats from impending ischemic damage of cortex if this treatment is delivered within the first two hours post-permanent middle cerebral artery occlusion (pMCAo). The current study sought to extend our findings in rats to mice, which would allow new avenues of research not available in rats. Thus, young adult C57BL/6J and CD1 mice were tested for protection from ischemic stroke with the same protective sensory stimulation-based treatment. Cortical activity and blood flow were assessed with intrinsic signal optical imaging (ISOI) and laser speckle imaging (LSI), respectively, and histological analysis (TTC) was performed at the completion of the experiments. Standing in stark contrast to the positive results observed in rats, in both strains we found that there were no differences between treated and untreated mice at 24 hours post-pMCAo in terms of infarct volume, negative functional imaging results, and major reduction in retrograde collateral blood flow as compared to pre-pMCAo baseline and surgical controls. Also, no differences were found between the strains in terms of theses variables. Potential reasons for the differences between rats and mice are discussed.

## Introduction

The search for a treatment for ischemic stroke has met many challenges. With stroke currently the fifth leading cause of death in the United States, second globally, and a leading cause of serious long-term disability [1,2], there is a need for the development of a rapid and long-lasting therapeutic that can benefit a large population of stroke patients. The difficulty with this task partially lies in the fact that the stroke population is heterogeneous, and many patients do not receive medical care before ischemia has damaged the brain. Our lab has previously reported that a sensory stimulation-based collateral therapeutic can completely protect rats from impending ischemic damage if delivered within the first 2 hours post-pMCAo [3]. This type of treatment, if relevant to humans, is promising as it is noninvasive, nonpharmacological, and requires no special equipment.

Earlier work from our lab has shown that in a rat model, the pial collateral vessels that anastomose with the distal MCA branches are critical to reperfusion of the occluded MCA and protection of ischemic cortex. When these distal branches were occluded in addition to the standard MCA occlusion at the M1 segment, rats were not protected despite having received immediate whisker stimulation [3]. Therefore, retrograde reperfusion of the MCA occurs through these existing patent collaterals, ensuring that the viability of neurons in the threatened region is maintained. Additionally, this protection has been observed under both pentobarbital and isoflurane anesthesia [4], and in awake, behaving subjects [5]. Since an important component underlying cortical protection by sensory stimulation has been elucidated (collateral blood flow), investigation of the underlying molecular mechanisms of protection was a logical next step.

The use of mice as animal models has flourished in recent decades due to the availability of genetic manipulations that enable the dissection of molecular mechanisms in models of disease. The C57BL/6J strain, widely used in stroke research, is known to have numerous pial collaterals [6,7,8], and in fact was shown to have high numbers of collaterals and larger collateral vessel diameters than 14 other mouse strains [9]. Additionally, Chalothorn et al. [10] has shown that CD1 mice, a slightly larger mouse strain, also have functioning collaterals.

Importantly, there is increasing evidence that poor pial collateral vessel flow in humans is a critical predictor of stroke severity as it has been linked to poor outcome even in the event of recanalization [11–16]. Thus, given the importance of pial collaterals for the effectiveness of our treatment, as well as for clinical outcome, our main goal was to test whether immediate delivery of the collateral-based sensory stimulation treatment used in our lab for rats, could also protect the C57BL/6J and CD1 mouse strains from impending ischemic stroke damage. If protected, these mice would open new research avenues for exploring the mechanisms underlying the protection we observe.

## Materials and methods

All procedures were in compliance with NIH guidelines and approved by UC Irvine Animal Care and Use Committee (protocol #: 1997–1608, assurance ID#: A3416.01), and in compliance with the ARRIVE guidelines.

### Subjects and surgical preparation

Twenty-five experimental subjects, 25-30g 10–12 week old male C57BL/6J mice (Jackson Laboratories, Bar Harbor, ME, USA), and twenty-four experimental subjects, 30-40g 10–12 week old male CD1 mice (Charles River Laboratories, Wilmington, MA, USA) were individually housed in standard cages. At the beginning of each experiment, subjects were injected intraperitoneally with a Nembutal bolus (50 mg/kg b.w.). Supplemental injections of Nembutal (27.5 mg/kg b.w.) were given as necessary. After resection of soft tissue, the parietal bone was thinned to ~150μm using a dental drill to create an ‘imaging’ area in the skull over the left primary somatosensory cortex. 5% dextrose (0.3mL) and atropine (0.05 mg/kg, b.w.) were administered at the beginning of the experiment and every six hours after until the animal was returned to its home cage. Body temperature was measured via a rectal probe, and maintained at 37° Celsius by a self-regulating thermal blanket. Auto clipping of the skin above the imaging window was performed 5 hours after pMCAo for all experimental groups. Animals were returned to their home cage and allowed to recover overnight prior to all +24 hour experimentation.

### Overview

Using a within subject design that is identical to our previous studies, 25 C57BL/6J mice and 24 CD1 mice were randomly assigned to a +0h group (+0h stands for immediate or zero hours following pMCAo), a no-stimulation control group, or a surgical sham group. Baseline functional imaging (ISOI) and blood flow imaging (LSI) was collected for all subjects at the beginning of surgery. All +0h subjects (n = 8, C57BL/6J; n = 8, CD1) then received a pMCAo, and immediate post-occlusion single whisker stimulation. Pre- and post-occlusion whisker stimulation consisted of 1 s of 5 Hz deflections of a single whisker (whisker C2). Post-occlusion, this stimulation treatment was intermittently (with random intervals averaging 21 seconds) delivered 256 times, totaling 4.27 minutes of stimulation over the course of 2 hours [3]. No-stimulation controls (n = 8, C57BL/6J; n = 8, CD1) underwent identical pMCAo to that of +0h subjects, but never received whisker stimulation; pMCAo was immediately followed by a 5-hour no-stimulation period. Surgical shams (n = 7, C57BL/6J; n = 8, CD1) underwent identical surgery to that of +0h subjects, with the suture needle and thread passing under the MCA, but sutures were not tied around the MCA, leaving the blood vessel intact. Sham surgery was immediately followed by whisker stimulation. +0h and sham subjects remained anesthetized throughout the 2-hour stimulation period, while no-stimulation controls remained anesthetized throughout the 5-hour no-stimulation period. Experiments were designed so animals were randomly assigned to one of the three groups after pMCAo. Additionally, two +0h subjects received full whisker array stimulation treatment according to the same stimulation paradigm. After whisker stimulation or quiet period, all mice were placed back in their home cage for recovery until their follow-up assessment at 24 hours post-pMCAo, which consisted of ISOI and LSI (animals receiving full whisker array stimulation only underwent ISOI). At the end of pMCAO surgery, mice received subcutaneous ampicillin antibiotic injections (100 mg/kg), and Flunixin meglumine analgesic was injected subcutaneously (2 mg/kg). The closed wound was covered with topical antibiotic, and mice were monitored while recovering from anesthesia. At the end of 24 hour imaging, mice were then euthanized with sodium pentobarbital (50 mg/kg, intraperitoneally), and the brains were harvested for histological assessment. All analysis was conducted under blinded conditions.

### Histology (2,3,5-triphenyltetrazolium chloride staining for infarct)

At the conclusion of each experiment, mice were euthanized and the brain was removed, sectioned into 2 mm coronal slices, and incubated in 2% 2,3,5-triphenyltetrazolium chloride at 7°C for 20 min in the dark [17]. The TTC-stained sections were photographed with a digital camera, and images were analyzed using ImageJ software. The total infarct volume is determined by multiplying the infarct area of each slice by the slice thickness. An observer blind to experimental condition performed this volume calculation. A small surgical lesion is occasionally apparent at the immediate site of MCA occlusion. This occurs infrequently and equivalently in all experimental groups. The small amount of damage occasionally produced at the surgical site can be readily distinguished from the large ischemic infarct and is excluded from infarct analysis [18].

### Permanent middle cerebral artery occlusion (pMCAO)

Permanent ischemic conditions were modeled after the same procedure in rats [19]. The base of the left proximal middle cerebral artery at the M1 segment [18,20,21] is permanently occluded, blocking flow to all MCA cortical branches. This is achieved by careful removal of the skull and dura from a 2x2mm ‘surgical window’ placed beyond the bottom left portion of the imaging window, directly over the M1 segment of MCA, just distal to MCA’s lenticulostriate branches and proximal to any cortical branching. The M1 occlusion therefore entails that only cortical ischemic damage is expected in this occlusion model. A half-curve reverse cutting suture needle is cut in half and threaded with two 4–0 silk threads and passed through the pial layer of the meninges, below MCA (the needle is kept above the cortical surface to the extent possible to minimize damage). Then the two threads (moved to ~1mm apart after being strung beneath the artery) are both tied and tightened around MCA and the vessel is transected (completely severed) between the two knots. Care is taken to avoid damaging the artery, and experiments are terminated if there are signs of bleeding from MCA (1 case).

### Intrinsic signal optical imaging (ISOI) and analysis

A detailed description of ISOI data acquisition and analysis can be found elsewhere [22–24]. Briefly, a charge coupled device (CCD) camera (either a 16-bit Cascade 512F or a 12-bit Quantix 0206, Photometrics, Tucson, AZ, USA) equipped with an inverted 50 mm AF Nikon lens (1:1:8, Melville, NY, USA) combined with an extender (model PK-13, Nikon, Melville, NY, USA) was used for imaging and controlled by V++ Precision Digital Imaging System software (Digital Optics, Auckland, NZ). During each 15-s trial, 1.5 s of prestimulus data followed by 13.5 s of post stimulus data is collected, with a 6±5 sec random inter-trial interval. Stimulus consists of a single whisker (or full whisker array) being deflected by 9° in the rostral-caudal direction at a rate of 5 Hz for a total stimulus duration of 1 second. The cortex is illuminated with a red light emitting diode (635 nm maximum wavelength). Data are collected in blocks of 64 stimulation trials, and a sampled time point (for example pre-pMCAo baseline) is considered complete upon summation of 128 stimulation trials. Ratio images are created from calculating fractional change (FC) values by dividing each 500ms frame of post-stimulus signal activity by the 500ms frame of pre-stimulus intrinsic signal activity collected immediately before stimulus onset. The ratio image containing the maximum areal extent for the intrinsic signal is Gaussian filtered (half width = 5) and the areal extent quantified at a threshold level of 2.5 x 10^−4^ fractional change away from zero. Peak amplitude is quantified in fractional change units from the pixel with the peak activity within the maximum areal extent.

### Laser speckle imaging (LSI) and analysis

A detailed description of LSI [25,26] data acquisition and analysis can be found elsewhere [3]. Briefly, a 632.8 nm 15 mW HeNe laser was used as the illumination source over a region of ~25mm^2^, and collected images were processed as previously described [3]. Speckle contrast images were converted to speckle index images by calculating their inverse squares multiplied by the exposure time in seconds, so that larger index values corresponded to faster blood flow. Speckle index images were then averaged to improve signal-to-noise ratio. To quantify blood flow within the MCA, we calculated the mean value within a region of interest (ROI) in MCA cortical branches as defined according to several criteria described previously [3]. All flow index values were scaled over a range where 0 flow was set at noise values. At the completion of 24 hours imaging, subjects were sacrificed with Nembutal, and dead animal (noise) values were collected ten minutes after cessation of the heart beat; these dead values were subtracted from all baseline and 24 hour values.

### Statistical analysis

For imaging data, ANOVA were run on baseline values in all experimental groups to ensure no significant differences before pMCAo. Because there were no responses to quantify at 24 hours, post-pMCAo imaging evoked area and amplitude were converted to difference score values (post-occlusion—baseline) with values away from 0 signifying a change from baseline. A constant was added to difference values, which were then transformed with a square root function to better satisfy the assumptions of ANOVA and inferential statistics were performed on the transformed data. For speckle imaging, average baseline arbitrary units measurements were normalized to 100%, and % change from baseline values were calculated for all measurements after pMCAo. For all experimental groups, following ANOVA, specific contrasts were calculated to identify which groups differed from baseline. Alpha level was set to 0.05 and Bonferroni adjustments were applied to account for multiple contrasts. Infarct volume comparisons were also calculated by employing ANOVA. All plotting and statistics were performed using SYSTAT 11 (SYSTAT Software Inc., Chicago, IL, USA).

## Results

### Treatment does not protect cortical activity in C57BL/6J or CD1 mice

**Fig 1:**
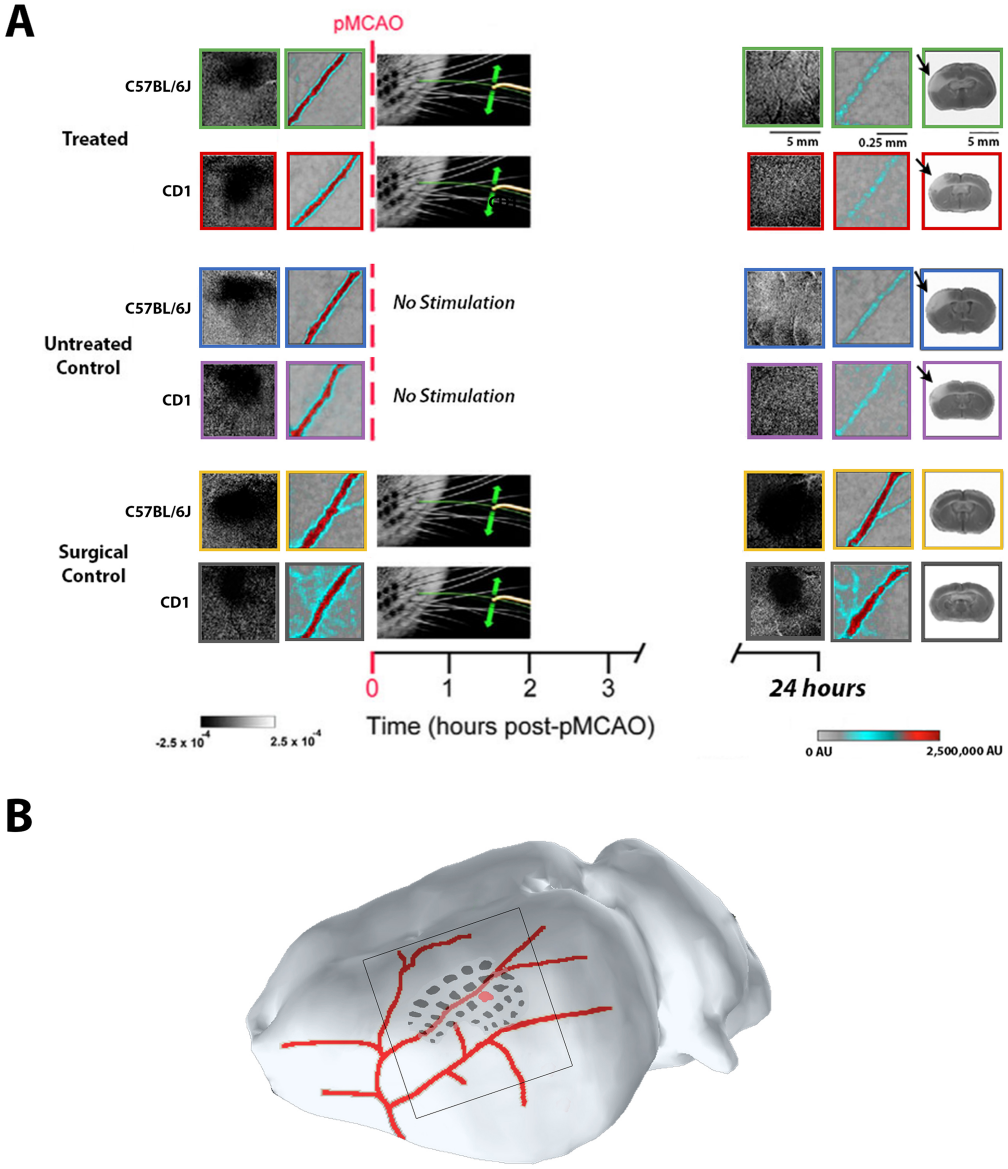
Treated C57BL/6J and CD1 mice are not protected from ischemic damage. A, Experimental schema with ISOI, LSI and TTC representative cases for C57BL/6J (green, blue, and yellow boxes) and CD1 mice (red, purple, and grey boxes): treated (top), untreated (middle), and surgical control (bottom) subjects, before (left) and 24 hours after (right) pMCAo. All subjects had whisker functional representations (WFRs) and blood flow within the MCA at baseline. When assessed at 24 hours post-pMCAo, treated and untreated subjects of both strains lacked WFRs, retrograde blood flow within the MCA was minimal, and TTC staining revealed infarct (right; see arrows). Surgical controls of both strains, however, maintained both WFRs and MCA blood flow, and did not sustain infarct since these subjects never received pMCAo. B, The imaging window (black-rimmed square) is centered over the barrel cortex (black regions), which is fed by major MCA branches (in red); the C2 whisker barrel is highlighted in red. The smaller representative images of LSI blood flow within the MCA are taken from a portion of a major MCA branch within this imaging window; the location in relation to C2 can vary between animals.

**Fig 2:**
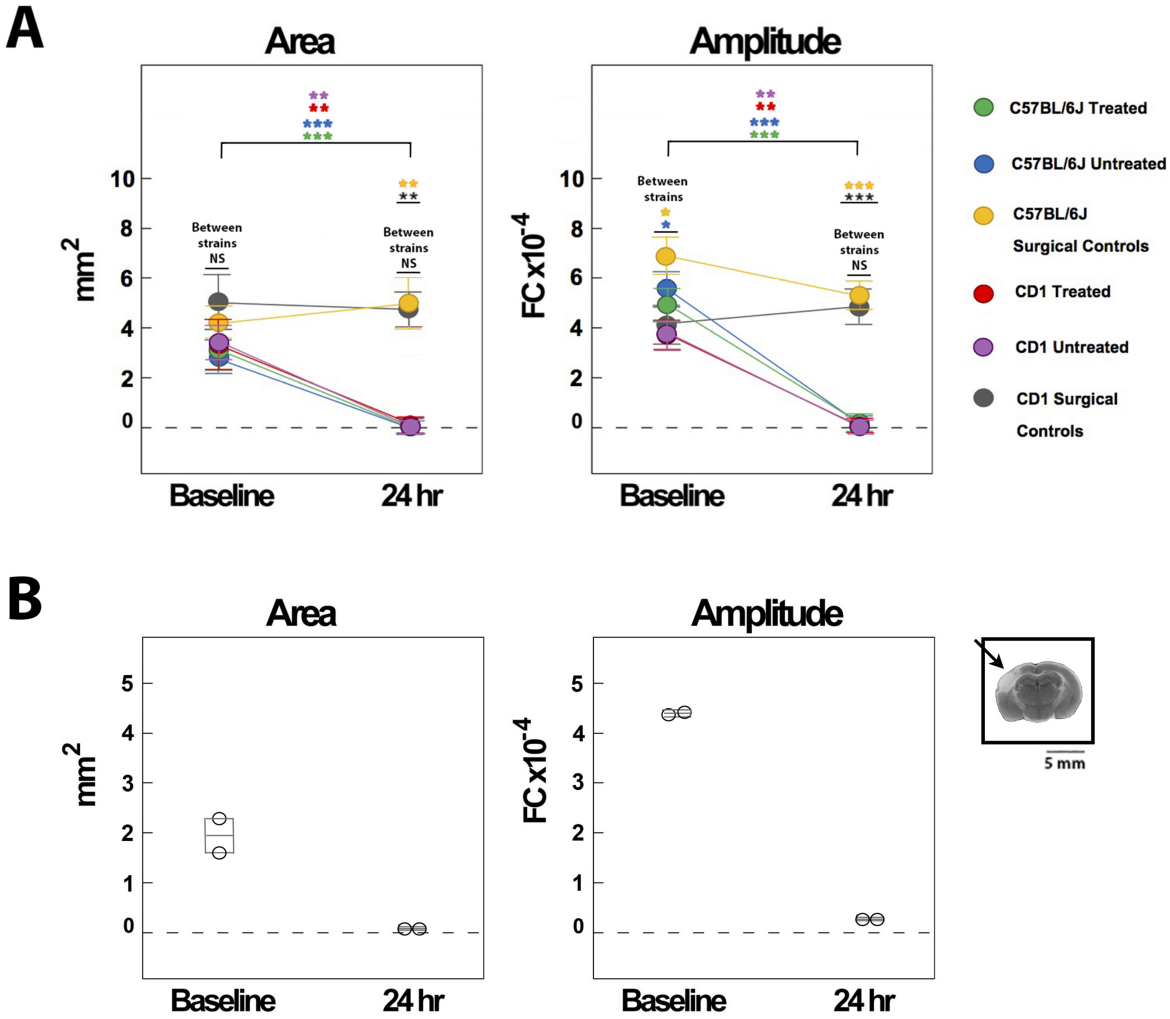
WFR quantification for C57BL/6J and CD1 mice. A, WFRs were quantified in terms of their area and amplitude at baseline and 24 hours post-pMCAo. WFR quantification for C57BL/6J and CD1 mice shows that treated and untreated subjects had significant reductions in WFRs at 24 hours, while surgical controls maintained cortical activity. No differences were found between treated, untreated, and surgical control subjects across C57BL/6J and CD1 strains, indicating these groups were equivalent at baseline and 24 hours. (NS = not significant, * = p ≤ 0.05, ** = p ≤ 0.01, *** = p ≤ 0.001). B, Two mice received full whisker array stimulation, and both subjects lacked WFR’s at 24 hours; TTC representative case shows infarct at 24 hours (black arrow).

Before pMCAO, there were no significant differences in the area or amplitude of the whisker functional representation (WFR) between the three C57BL/6J groups, or between the three CD1 groups (n = 8 for all groups). However, at 24 hours post-pMCAo there was a significant difference between C57BL/6J groups for both area (F_2,20_ = 6.26, P < 0.01, ANOVA) and amplitude (F_2,20_ = 7.46, P < 0.001, ANOVA). Post-hoc Tukey’s HSD tests showed that the area and amplitude for surgical controls (n = 7) were significantly different from the other two groups at 24 hours. Despite receiving immediate treatment, the treated C57BL/6J mice (n = 8) were equivalent to untreated C57BL/6J subjects (n = 8), with both groups exhibiting a reduction in both area and amplitude at 24 hours compared to baseline. For treated subjects, this reduction was significant for both area and amplitude (area: F_1,20_ = 10.85, P < 0.005; amplitude: F_1,20_ = 41.54, P < 0.001), and the same was true for untreated subjects (area: F_1,20_ = 9.96, P < 0.005; amplitude: F_1,20_ = 59.49, P < 0.001). Surgical controls, although trending for an increase in area, didn’t have a significant change in area or amplitude at 24 hours post-pMCAo (area: F_1,20_ = 0.51, P > 0.05; amplitude: F_1,20_ = 4.49, P < 0.05, not significant with Bonferonni correction) (Figs 1A and 2A).

Similar results were found for CD1 mice. At 24 hours post-pMCAO, there was a significant difference between CD1 groups for both area (F_2,21_ = 6.84, P < 0.01, ANOVA) and amplitude (F_2,21_ = 10.02, P < 0.001, ANOVA). Post-hoc Tukey’s HSD tests showed that the area and amplitude for surgical controls were significantly different from the other two groups at 24 hours; CD1 treated and untreated subjects showed a drastic reduction in area and amplitude at 24 hours post-pMCAo. This reduction was significant for both area and amplitude for treated (area: F_2,21_ = 6.21, P < 0.01; amplitude: F_2,21_ = 13.88, P < 0.01) and untreated subjects (area: F_2,21_ = 14.02, P < 0.01; amplitude: F_2,21_ = 15.41, P < 0.01) (Figs 1A and 2A).

Finally, we determined whether there were any differences between the three groups across both C57BL/6J and CD1 strains. At baseline, we observed no difference among experimental groups or strains for area. For amplitude, there was no difference among experimental groups, but there was for strains (untreated: F_1,41_ = 6.42, P < 0.02, ANOVA; surgical controls: F_1,41_ = 5.62, P < 0.02, ANOVA). At 24 hours post-pMCAo, there was a difference among experimental groups for area (F_2,41_ = 25.86, P < 0.01, ANOVA) and amplitude (F_2,41_ = 49.31, P < 0.001, ANOVA), since surgical controls maintained baseline levels of evoked activity but treated and untreated controls had significant reductions. There was no difference among strains, however, for either area or amplitude (Fig 2A).

Full whisker array stimulation was tested in two C57BL/6J mice and, similar to subjects receiving single whisker stimulation, neither subject was protected according to ISOI and TTC analysis (Fig 2B).

### Mice exhibit minimal collateral-based blood flow to the MCA post-occlusion

**Fig 3:**
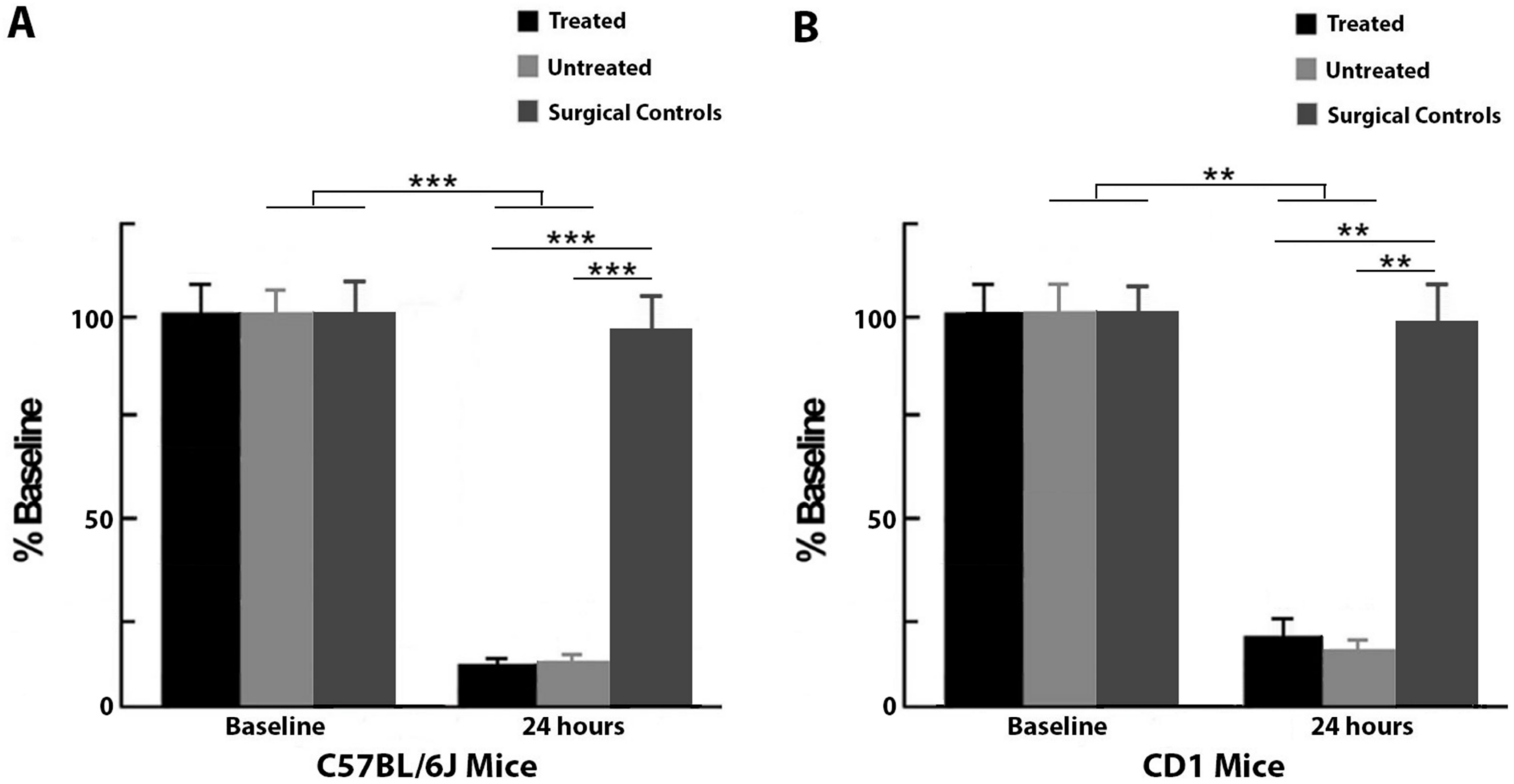
Blood flow quantification for C57BL/6J and CD1 mice at baseline and 24 hours post-pMCAo as a percent of baseline flow. A, At baseline, C57BL/6J treated, untreated and surgical control groups were equivalent, but at 24 hours post-pMCAO, treated subjects were equivalent to untreated subjects and had significantly reduced blood flow within the MCA. Surgical controls maintained baseline levels of flow at 24 hours. B, Similar to C57BL/6J mice, CD1 treated and untreated subjects were equivalent and had significant reductions in blood flow at 24 hours, while surgical controls maintained baseline levels of flow at this time point. (** = p ≤ 0.01, *** = p ≤ 0.001).

C57BL/6J and CD1 mice underwent laser speckle imaging to assess whether there was retrograde blood flow in the MCA 24 hours post-occlusion. While subjects from all C57BL/6J groups had similar blood flow at baseline, there was a significant difference between groups at 24 hours post-pMCAo (F_2,20_ = 14.53, P < 0.001, ANOVA). Post-hoc Tukey’s HSD test showed that blood flow in surgical controls was significantly different from the other two groups at 24 hours. After pMCAo, both treated and untreated subjects showed a severe reduction in blood flow within the MCA, indicating minimal collateral-based blood flow. Treated (n = 8; F_1,20_ = 22.31, P < 0.001) and untreated (n = 8; F_1,20_ = 22.24, P < 0.001) C57BL/6J mice had significantly lower values compared to surgical controls (n = 7). (Figs 1A, 1B and 3A).

Similar to the C57BL/6J mice, there was a significant difference in blood flow between CD1 groups at 24 hours post-occlusion (F_2,21_ = 8.62, P < 0.005, ANOVA), and post-hoc Tukey’s HSD test showed that the surgical controls (n = 8) were significantly different from treated and untreated subjects. Treated (n = 8; F_1,21_ = 12.65, P < 0.005) and untreated (n = 8; F_1,21_ = 13.21, P < 0.005) groups both had major reductions in MCA blood flow at 24 hours post-pMCAo (Figs 1 and 3B).

### Histology revealed that mice sustain ischemic damage despite receiving immediate treatment

**Fig 4:**
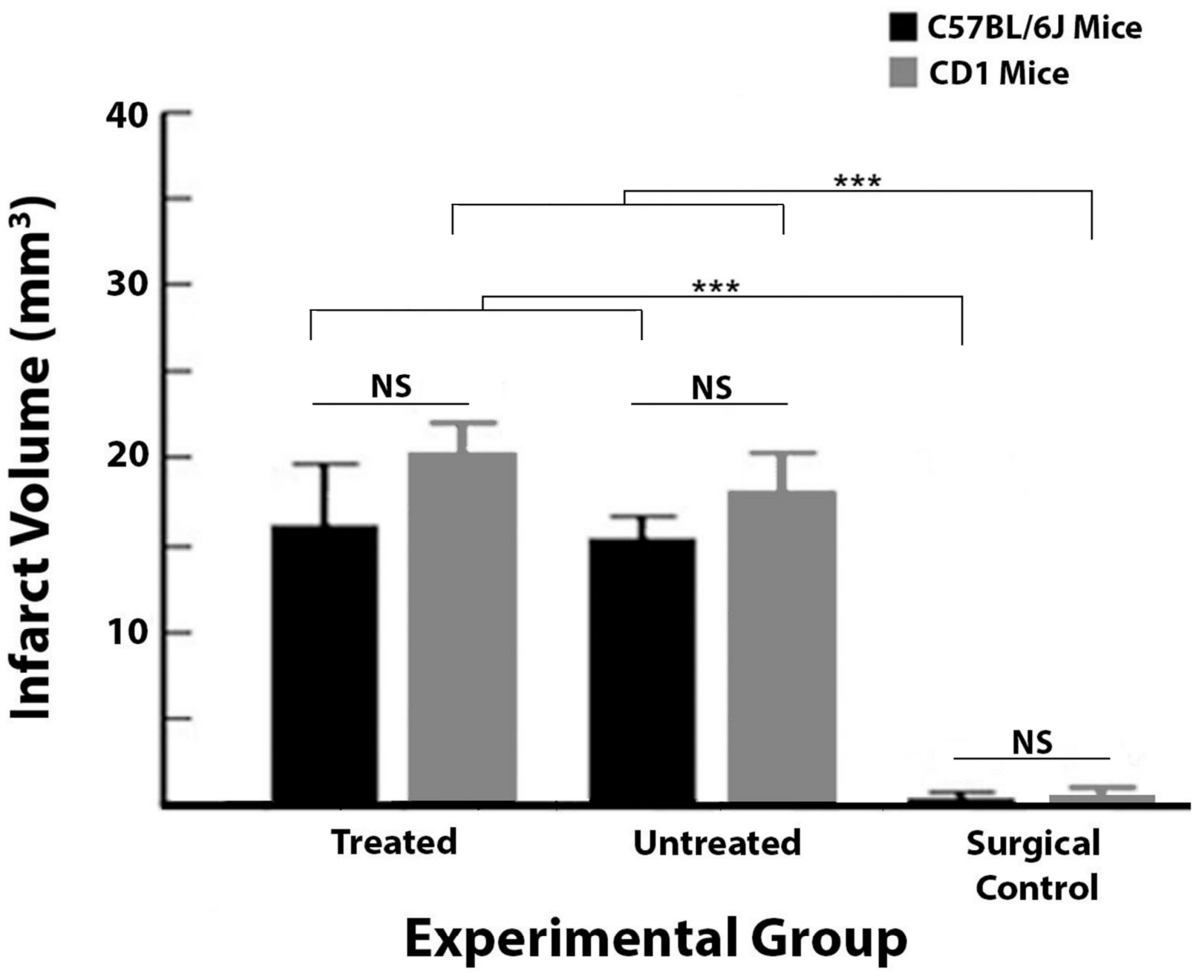
TTC staining revealed no protective effect of treatment for either C57BL/6J or CD1 subjects. Infarct quantification for C57BL/6J and CD1 treated, untreated and surgical control groups. Despite receiving immediate treatment, treated subjects sustained infarct equivalent in size to untreated subjects. Surgical controls were significantly different from treated and untreated subjects as they did not sustain ischemic damage, but occasionally subjects would have evidence of surgical damage that was less than 1 mm^3^. Additionally, C57BL/6J and CD1 groups were equivalent for infarct volume. (NS = not significant, *** = p ≤ 0.001).

Ischemic damage was assessed with TTC staining and revealed that immediate treatment did not prevent infarct in C57BL/6J or CD1 mice (Figs 1A and 4). There was a significant difference in infarct size between C57BL/6J groups (treated: n = 8; untreated: n = 8; surgical controls: n = 7, no infarcts but occasionally there would be evidence of surgical damage of <1mm^3^; F_2,20_ = 18.45, P < 0.001, ANOVA), and post-hoc Tukey’s test showed the significant difference lied only between surgical controls and the other two C57BL/6J groups. For treated and untreated subjects, this is 6.93 ± 1.23% and 6.94 ± 0.52% of the ipsi-ischemic hemisphere, respectively. Thus, treated and untreated C57BL/6J groups sustained infarcts of equivalent size, while the surgical controls had no ischemic damage since they did not receive pMCAo.

A significant difference was observed between CD1 groups (F_2,19_ = 37, P < 0.001, ANOVA); post-hoc Tukey’s test showed that CD1 surgical controls (n = 8) were significantly different from treated (n = 8) and untreated control groups (n = 6).

When represented as a percentage of ipsi-ischemic hemisphere, treated, untreated and control subjects had infarcts of 9.36 ± 1.02%, 8.37 ± 1.12%, and 0.48 ± 0.20% respectively. Finally, when comparing treated, untreated and surgical control groups for both C57BL/6J and CD1 strains, there was an effect of group since surgical controls from neither strain sustained infarct (F_2,39_ = 45.70, P < 0.001, ANOVA), but importantly, there was no difference in infarct size between groups among the two strains.

## Discussion

This study assessed whether our previous findings of complete protection from impending ischemic stroke damage in rats could be replicated in an additional species. Mice were chosen since, if findings had been replicated, this would allow us to begin dissecting the molecular mechanisms underlying this treatment. Although both C57BL/6J and CD1 mice are known to have functioning collaterals, we did not observe protection from ischemic damage in either strain with this collateral-based single-whisker stimulation treatment. Additionally, subjects that received full-whisker array stimulation treatment were not protected from ischemia either. Despite receiving whisker stimulation treatment immediately post-occlusion, treated and untreated subjects from both strains exhibited significant reductions in cortical activity and blood flow 24 hours post-pMCAo, while C57BL/6J surgical controls confirmed that the surgical manipulations themselves (aside from pMCAo) were not responsible for the impairments observed. Ischemic damage was assessed histologically with TTC staining, confirming the presence of infarct in untreated and treated subjects in both mouse strains. Notably, the presence of the stimulation treatment didn’t change the outcome. It is helpful to think about infarct size in these mice in terms of percent infarct of the ipsi-ischemic hemisphere so comparisons can be made to our previous studies in rats. The percentages presented here in mice fall within the range of what we have observed in our rat studies (3–12% of hemisphere) [19], and they are also comparable to what is observed in humans (4.5–14% of hemisphere) [27].

The minimal retrograde MCA blood flow observed here suggests that the main potential problem may be that pial collaterals in these mice are not recruited in the same manner as in rats that do show protection when receiving immediate treatment [28]. In these mouse strains, the significantly reduced blood flow at 24 hours post-pMCAo may not be sufficient to protect cortex from impending ischemic damage. It’s important to keep in mind that laser speckle blood flow images the surface vasculature, thereby limiting our ability to determine the role of any additional factors. Thus, in addition to the possibility of impaired pial collateral function, we can postulate that the lack of sufficient blood flow and protection from ischemic stroke could be due to several factors, such as an incomplete circle of Willis, impaired neurovascular coupling and functional hyperemia, and/or impaired function of penetrating arterioles. These will be discussed further below.

Although pial collaterals have been shown to be critical to reducing infarct size, the circle of Willis in C57BL/6J mice is known to be highly variable in terms of the extent of its primary collateralization [29], with many mice lacking either one or both posterior communicating arteries, leaving about 10% with a complete circle of Willis [30]. It’s important to note here that this percentage is much smaller than the percentage of humans with a complete circle of Willis, and that rats are similar to humans in this respect [21,31]. Thus, the reduced redundancy in blood flow at this early level of cerebral vascularization in these mice may set the stage for the decreased ability to sustain sufficient pial collateral flow after an ischemic event. Additionally, C57BL/6J mice are known to have fewer pial collaterals between the PCA and MCA compared to collaterals interconnecting the ACA and MCA trees [9]. It is not clear from our previous work in rats whether collaterals from the ACA or PCA trees are involved to an equal extent or whether one is more involved than the other in protection from ischemic damage. If collaterals from the PCA are recruited to a larger extent than those from the ACA, this could help explain the lack of protection in mice, however further work is necessary to determine the involvement of collaterals from different arterial trees.

Despite this deficit, there is evidence that these mice do have some pial collateral flow after MCA occlusion. As Zhang et al. [9] showed, C57BL/6J mice had large numbers of pial collaterals and larger collateral vessel diameters compared to fourteen other mouse strains, and this was associated with smaller infarcts than in other strains. Similarly, Li and Murphy [32] observed spontaneous retrograde flow in pial collaterals of C57BL/6J mice from the Anterior Cerebral Arterial (ACA) tree 19 minutes after a temporary filament occlusion of the MCA, however, this flow decreased closer to the core of the MCA tree and this damaged region did not recover after reperfusion as a result of removal of the occlusion. Thus, despite the presence of spontaneous reperfusion via pial collaterals in both of these studies, it was not sufficient to completely protect from ischemic damage under their conditions. Additionally, Cristofaro et al. [33] found that transgenic CD1 mice that had increased density of pial collaterals also sustained ischemic damage, as not all collaterals were functional. It becomes clear from these results, along with our data, that the impact of collateral flow on infarct size, or complete protection from ischemic damage in the case of our rat studies, relies not just on collateral vessel numbers but importantly on vessel functionality.

Another potential explanation for the lack of protection is perhaps that the mechanisms of neurovascular coupling that mediate the functional hyperemia response differ between rats and mice. Spontaneous reperfusion occurs as a result of pressure drop after MCA occlusion, which leads to the pial collateral anastomoses dilating to allow retrograde flow from the ACA and PCA [9,16,34], thereby potentially reducing infarct size but not resulting in complete protection from damage. However, functional hyperemia during evoked cortical activity results in dilation of the local vasculature in order to meet the increased energy demands of the tissue. Sensory stimulation treatment takes advantage of functional hyperemia, resulting in enhanced collateral flow. Although we have shown that enhanced collateral flow has resulted in complete protection from ischemic stroke in rats [3–5,28,35–36], this enhanced flow was not observed in mice. Neurovascular coupling is known to be impaired to varying degrees under ischemic conditions, and targeting these coupling mechanisms has been suggested as a strategy for reducing damage that may succeed in translation to humans [37]. Thus, it would not be too surprising if an impaired coupling response was responsible for the lack of protection in mice. Further studies are necessary to determine to what extent functional hyperemia and pial collateral dilation may be impaired during ischemia in order to understand the inability of the vasculature in these mice to respond to our collateral-based sensory stimulation treatment in a similar manner as in rats.

Related to the idea of impaired neurovascular coupling and functional hyperemia, it’s also important to consider that the penetrating arterioles may not have dilated sufficiently to feed the capillaries, and thus the parenchyma [38,39]. Penetrating arterioles can be thought of as bottlenecks, and their responsiveness has been shown to ultimately regulate the rescuing of penumbral tissue since there is no blood flow between neighboring penetrating arterioles as they are not interconnected. Additionally, their dilation is associated with dilation of upstream pial vessels [40] and, in fact, the dilation initiated by active neurons can be propagated retrogradely to pial arterioles [41,42]. In C57BL/6J mice, Baran et al. [38] showed that penetrating arterioles connected to the MCA that are close to numerous pial collaterals between MCA and ACA dilate, whereas those penetrating arterioles further away from collaterals constrict. They concluded that to have dilation of penetrating arterioles to support protection of the tissue from ischemia, there must be blood flow in the pial collaterals through the anastomoses between the MCA and ACA. The coupling of the dilation of pial collaterals and penetrating arterioles after ischemic stroke is clearly a complex process. In our model, it is possible that the evoked cortical activity from treatment did not result in adequate dilation and retrograde blood flow through the pial collaterals, leading to impaired dilation of penetrating arterioles, further compounding the issue and resulting in infarct. Our LSI results would also support this interpretation since minimal blood flow would be observed in MCA if penetrating arterioles were not adequately dilated. In future studies, it would be important to assess perfusion in the penetrating arterioles, perhaps with functional ultrasound [43], in order to dissect their role in protection from or deterioration to damage.

The difference in outcome between rats and mice begs the question of which one is a better model for humans. We believe that both can be relevant models and can represent different stroke patient populations. Significant differences in the amount and functionality of cerebral collateralization are documented in humans [9,12,14,44,45]. Thus, our rat studies may represent humans that have well-developed functional pial collaterals and less impaired neurovascular coupling, while results from the mice may indicate the potential outcome for stroke patients that lack functioning collaterals without comorbidities. If relevant to humans, the collateral-based sensory stimulation treatment described by our lab may be a promising treatment for some populations of ischemic stroke patients. In terms of translation of this treatment to humans, whiskers comprise a large portion of somatosensory cortex in rodents, with the equivalent in humans being the fingers and mouth. Both cortical regions in rodents and humans are fed by the MCA, thus, ischemic stroke within the MCA in humans could potentially be treated with stimulation of the hands or mouth. However, determination of the extent of cerebral collateralization in patients, along with the location of the stroke, should be routinely performed prior to administration of this treatment. This would not only confirm that the stroke has occurred in regions of the brain that can be treated with this mode of stimulation, but could also indicate which patients may show a beneficial response to our collateral based therapeutic treatment.
